# Pharmacological inhibition of MyD88 homodimerization counteracts renal ischemia reperfusion-induced progressive renal injury in vivo and in vitro

**DOI:** 10.1038/srep26954

**Published:** 2016-06-01

**Authors:** Li-Min Zhang, Jian-Hua Liu, Cheng-Biao Xue, Ming-Qiang Li, Shuai Xing, Xue Zhang, Wen-Tao He, Feng-Chao Jiang, Xia Lu, Ping Zhou

**Affiliations:** 1 Institute of Organ Transplantation, Tongji Hospital, Tongji Medical College, Huazhong University of Science and Technology; Key Laboratory of Organ Transplantation, Ministry of Health, and Key Laboratory of Organ Transplantation, Ministry of Education, Wuhan 430030, China; 2 Institute of Hepatobiliary Diseases of Wuhan University, Zhongnan Hospital of Wuhan University; Transplant Center of Wuhan University, Hubei Key Laboratory of Medical Technology on Transplantation, Wuhan 430071, China; 3 The Central Hospital of Taian, Taian 271000, China; 4 Department of Endocrinology, Tongji Hospital, Tongji Medical College, Huazhong University of Science and Technology, Wuhan 430030, China; 5 Academy of Pharmacology, Tongji Medical College, Huazhong University of Science and Technology, Wuhan 430030, China

## Abstract

The activation of innate immunity via myeloid differentiation factor 88 (MyD88) contributes to ischemia reperfusion (I/R) induced acute kidney injury (AKI) and chronic kidney injury. However, since there have not yet been any effective therapy, the exact pharmacological role of MyD88 in the prevention and treatment of renal ischemia reperfusion injury (IRI) is not known. We designed a small molecular compound, TJ-M2010-2, which inhibited MyD88 homodimerization. We used an established unilateral I/R mouse model. All mice undergoing 80 min ischemia through uninephrectomy died within five days without intervention. However, treatment with TJ-M2010-2 alone significantly improved the survival rate to 58.3%. Co-treatment of TJ-M2010-2 with the CD154 antagonist increased survival rates up to 100%. Twenty-eight days post-I/R of 60 min ischemia without nephrectomy, TJ-M2010-2 markedly attenuated renal interstitial and inhibited TGF-β1-induced epithelial-mesenchymal transition (EMT) of renal tubular epithelial cells. Furthermore, TJ-M2010-2 remarkably inhibited TLR/MyD88 signaling *in vivo* and *in vitro*. In conclusion, our findings highlight the promising clinical potential of MyD88 inhibitor in preventing and treating acute or chronic renal I/R injuries, and the therapeutic functionality of dual-system inhibition strategy in IRI-induced AKI. Moreover, MyD88 inhibition ameliorates renal I/R injury-induced tubular interstitial fibrosis by suppressing EMT.

Renal ischemia reperfusion injury (IRI) occurs in many pathogenic events, for instance, during kidney transplantation (KT), and induces acute and chronic injuries, including delayed graft function, decreased survival rate of the graft, chronic rejection, renal fibrosis and prolonged patient hospital stay^1^,^2^,^3^,^4^. There have not yet been any effective therapy that completely cure renal IRI, likely because it is so difficult to relieve kidney IRI-associated vascular and tubular injury, inflammation, intracellular events and tubulointerstitial fibrosis^4^,^5^,^6^. Thus, it is necessary to develop treatments that can reduce IRI and to explore efficient drug candidates to ameliorate graft dysfunction during KT.

Toll-like receptors (TLRs) are germline-encoded pattern recognition receptors that are most well known in the innate immune response, as they recognize pathogen-associated molecular patterns (PAMPs) and danger-associated molecular patterns (DAMPs)^7^,^8^,^9^. As renal ischemia reperfusion (I/R) is caused, the TLR-DAMP interaction is a key step in I/R pathogenesis^10^. Some DAMPs, for example, high-mobility group box 1^11^ and heat shock protein^12^, are released from intracellular compartments and play essential roles during renal I/R. Thus, TLR activation is required for downstream signaling events. TLRs are expressed in various cell types, including dendritic cells (DCs)^13^, B cells^14^ and kidney tubular epithelial cells^15^. Myeloid differentiation factor 88 (MyD88) is widely used by all TLRs except TLR3 and activates the mitogen-activated protein kinases (MAPKs) and the translocation of nuclear factor-κB (NF-κB) from cytosol to nucleus to induce various inflammatory cytokines, for example, interleukin-1β (IL-1β), interleukin-6 (IL-6) and tumor necrosis factor-α (TNF-α)^16^. Thus, TLR/MyD88 signaling is a key mediator of renal IRI induction. Furthermore, MyD88 is a potential target for preventing and treating renal IRI.

We designed and synthesized a series of small compounds, 2-aminothiazole derivatives, named TJ-M2010 (WIPO Patent Application Number: PCT/CN2012/070811), based on the MyD88 Toll/interleukin-1 receptor (TIR) domain. In this study, we focused on the specific effects of compound TJ-M2010-2 in renal IRI. TJ-M2010-2 alone exhibited powerful protective effects against IRI induced acute kidney injury (AKI) and chronic kidney injury (CKI). The dual system inhibition strategy by using our MyD88 inhibitor (TJ-M2010-2) against the innate immune system and CD154 antagonist (MR1) against the adaptive immune system showed even more powerful protection. Moreover, because epithelial-mesenchymal transition (EMT) plays a role in renal interstitial fibrosis, we suggest that MyD88 inhibition attenuates renal fibrosis by blocking EMT.

## Results

### TJ-M2010-2-MyD88 TIR domain interactions

**Figure 1:**
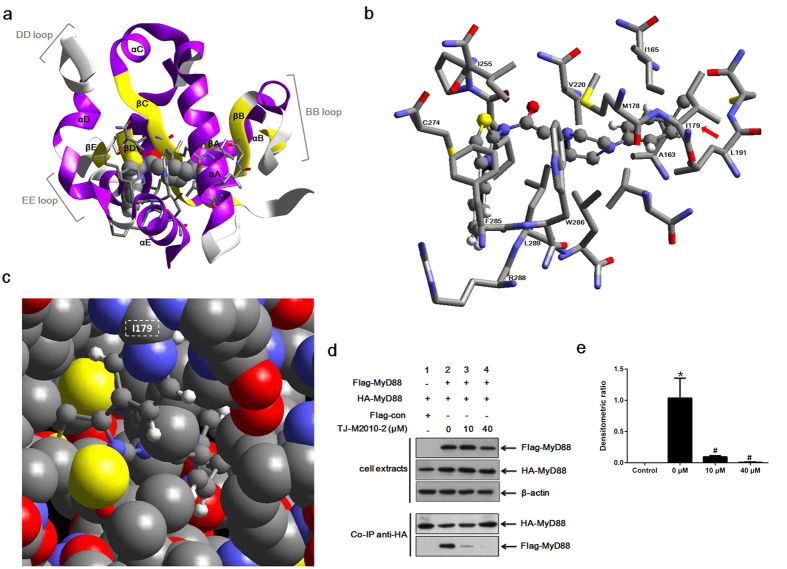
TJ-M2010-2 interacts with MyD88 TIR domain. (**a**) The crystal structure of most TIR domains contains a five-stranded β-sheet (**a–e**) surrounded by five α-helices (**a–e**). TJ-M2010-2 embedded into the MyD88 TIR domain and interacting with the αA, αE, βC, βD, DD loop and EE loop. The MyD88 TIR domain crystal structure based on the Protein Data Bank (PDB ID: 4DOM). The non-bond interaction score was −747.325 kcal/mol. (**b**) TJ-M2010-2 acted on the indicated MyD88 TIR domain amino acid residues. The red arrow indicates the Poc residue site I179. (**c**) The dominating interacting site I179 is magnified and marked by the white rectangle with dotted lines. Gray: carbon atom; blue: nitrogen atom; red: oxygen atom; white: hydrogen atom; yellow: sulfur atom. (**d**) Co-immunoprecipitation assay (Co-IP) of MyD88. HEK293T cells were co-transfected with HA-MyD88/Flag-MyD88 or HA-MyD88/Flag-con. Seven hours after transfection, TJ-M2010-2 was added to the medium. Total protein was extracted 48 hours after transfection. Proteins were incubated with anti-HA antibody and Protein A + G Agarose, and Flag-MyD88 bound to HA-MyD88 was detected by an anti-Flag antibody. As the concentration of TJ-M2010-2 increased (0 μM, 10 μM, 40 μM), the binding capacity between the Flag-MyD88 and HA-MyD88 proteins decreased (Co-IP, lane 2, 3, 4) (one of three independent experiments). (**e**) Densitometric analysis of Co-IP assays. The inhibition of dimerization of Flag- and HA- MyD88 proteins was dose-dependent, as 91% inhibited at 10 μM TJ-M2010-2 and 99% inhibited at 40 μM compared to 0 μM. The density of each HA-MyD88 lane was divided by that of Flag-MyD88 (*p < 0.01 versus Control; ^#^*p* < 0.01 versus 0 μM). Results are expressed as mean ± s.d. Control: lane 1 in Co-IP; 0 μM: lane 2 in Co-IP; 10 μM: lane 3 in Co-IP; 40 μM: lane 4 in Co-IP.

Molecule docking results demonstrated that TJ-M2010-2 localized to the 3D region constructed by box 1, box 3 and αA of the MyD88 TIR domain without the participation of box 2 (Box 1, a portion of the βA strand; box 2, the second part of the BB loop; box 3, the first part of the αE helix^17^). TJ-M2010-2 interacted with the residues of αA, αE, βC, βD, DD loop and EE loop but not with residues in the BB loop (Fig. 1a). As shown in Fig. 1b, there were 15 interacting sites that TJ-M2010-2 acted with MyD88 TIR domain. The benzene ring of the TJ-M2010-2 benzyl acted on I179, Y187, M178, and I182 of αA, W286 and L193 of αE, V220 of βC (piperazidine ring), I165 and A163 of βA, and L191 of βB (benzene ring) by hydrophobic interactions. The aminothiazole benzene ring of TJ-M2010-2 interacted with F285, L289, and R288 of αE. The TJ-M2010-2 aminothiazole ring acted on I255 of βD and C274 of the DD loop (Y187, I182 and L193 are not shown because they overlap other structures).

Although the BB loop is essential for MyD88 dimerization, our results showed that TJ-M2010-2 interacted with I179 (Poc site residue adjacent to BB loop) through hydrophobic interactions, disrupting its interactions of I179 with L199 and T202, which play a key role in sustaining BB loop stability (Fig. 1c). Therefore, TJ-M2010-2 indirectly targets the BB loop. A much more mobile BB loop made the TIR:TIR domain association unstable^18^.

### TJ-M2010-2 inhibits MyD88 homodimerization

To investigate how TJ-M2010-2 inhibits MyD88, we performed Co-immunoprecipitation (Co-IP) to analyze TJ-M2010-2-MyD88 interactions. As shown in Fig. 1d,e, TJ-M2010-2 treatment dose-dependently inhibited MyD88 homodimerization. Quantitative analysis showed that TJ-M2010-2 partially inhibited MyD88 homodimerization at 10 μM and completely inhibited MyD88 homodimerization at 40 μM.

### TJ-M2010-2 alone or with MR1 improves survival rate, prevents renal dysfunction and attenuates pathologic damage after IRI

**Figure 2:**
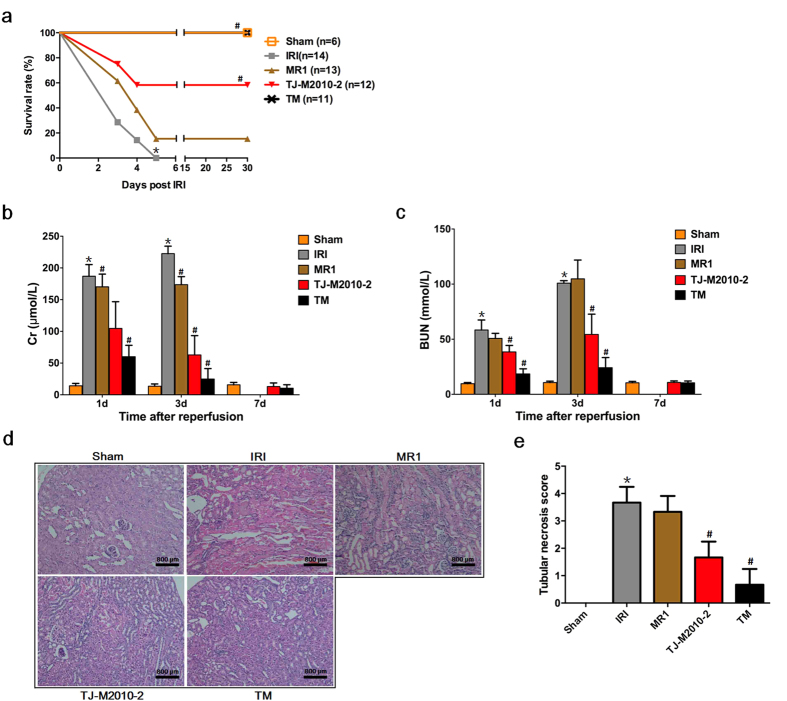
TJ-M2010-2 alone or in combination with MR1 prolongs survival, protects the renal function, attenuates pathologic damage of mice subjected to renal ischemia reperfusion injury (IRI). Mice were exposed to IRI for 80 min with uninephrectomy. (**a**) After the left renal pedicle was clamped for 80 min, mouse survival status was monitored for 30 days. The survival rate was 100% in Sham; 0% in IRI; 15.4% in MR1; 58.3% in TJ-M2010-2; 100% in TM (*p < 0.01 versus Sham; ^#^*p* < 0.01 versus IRI). (**b,c**) Six mice were sacrificed for each group. Blood samples were collected on day 1, day 3 and day 7 after reperfusion to measure serum creatinine (Cr) and blood urea nitrogen (BUN) levels (*p < 0.01 versus Sham; ^#^*p* < 0.01 versus IRI). Results are expressed as mean ± s.d. (**d**) Renal tissues were collected one day after IRI and stained with Hematoxylin & Eosin (three mice were sacrificed for each group). Original magnification × 200 over five fields. Histological sections of renal tissues are shown. Bar = 800 μm in all panels. (**e**) Histogram of tubular necrosis scores (*p < 0.01 versus Sham; ^#^*p* < 0.01 versus IRI). Results are expressed as mean ± s.d.

To investigate the therapeutic effects of TJ-M2010-2 on IRI induced acute and chronic injury, we used different IRI mouse models. As shown in Fig. 2a, all mice from IRI group died within five days without intervention. Strikingly, TJ-M2010-2 treatment alone resulted in a significantly higher survival rate of 58.3%. Interestingly, the dual system inhibitor (TJ-M2010-2 plus MR1) increased the survival rate to as high as 100%, as all of the mice survived 28 days after surgery. Furthermore, serum creatinine (Cr) and blood urea nitrogen (BUN) decreased to normal levels by seven days post-IRI after TJ-M2010-2 or dual system inhibitor treatment (Fig. 2b,c). However, there was no significant difference in serum Cr and BUN levels between the IRI and TJ-M2010-2 groups on day 28 (see Supplementary Fig. S1). We observed severe acute tubular damage in the cortex and medulla of kidneys from the IRI group on day 1, primarily seen as tubular necrosis and cast formation (Fig. 2d). Though the degree of damage was reduced in the MR1 group, there was significantly less damage in the TJ-M2010-2 and TM groups (Fig. 2e). The above results demonstrate that TJ-M2010-2 robustly protects against renal IRI.

### TJ-M2010-2 or dual system inhibition ameliorates inflammatory responses after IRI

**Figure 3:**
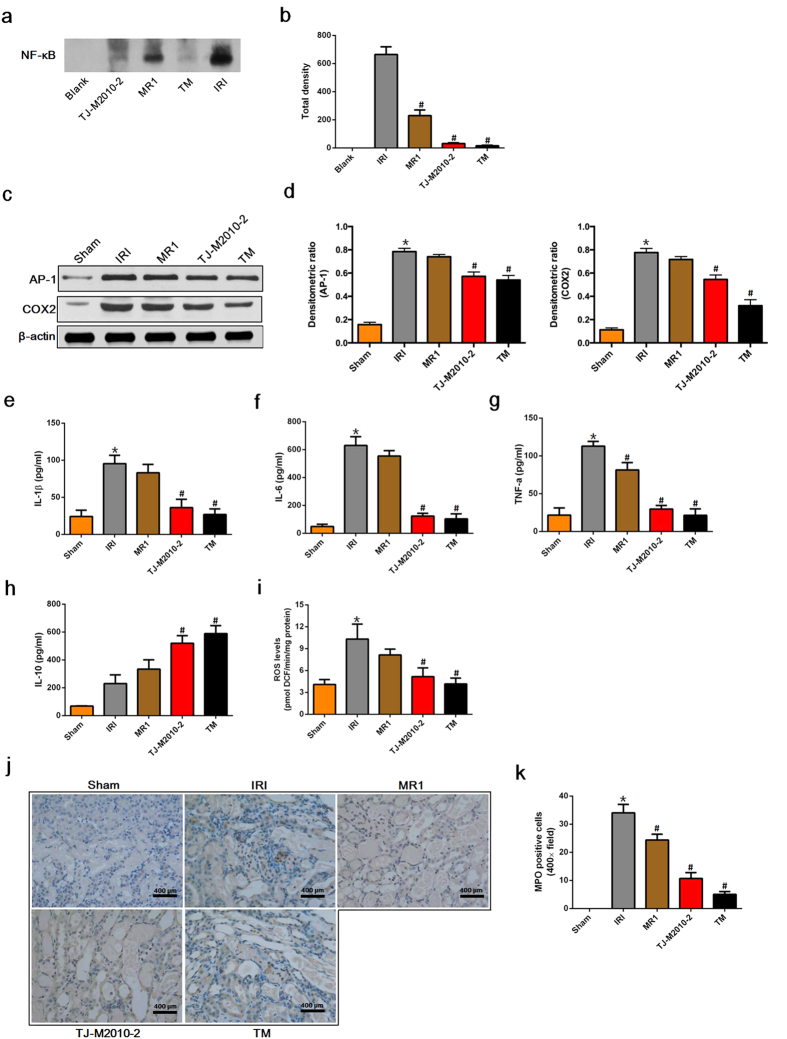
TJ-M2010-2 alone or with MR1 attenuates inflammatory responses after IRI. Mice were exposed to IRI for 80 min with uninephrectomy. (**a**) Nuclear proteins were extracted from kidney tissues one day after renal IRI and incubated with an NF-κB probe for 25 min (three mice were sacrificed for each group). EMSA assay was used to detect NF-κB activity (one of three independent experiments). (**b**) Densitometric analysis of the NF-κB band in EMSA. (^#^*p* < 0.01 versus IRI). Results are expressed as mean ± s.d. Blank: no protein was added. (**c**) Total proteins were extracted from renal tissues one day after IRI. AP-1 and COX2 expression were measured by Western Blot. (one of three independent experiments). (**d**) Densitometric analysis of Western Blot results. The density of each β-actin lane was divided by that of AP-1 and COX2 (*p < 0.0001 versus Sham; ^#^*p* < 0.0001 versus IRI). Results are expressed as mean ± s.d. (**e–h**) Serum samples were collected on day 1 after IRI (three of six mice sacrificed for the measurement of renal function for each group). IL1β, IL6, TNF-α and IL10 serum levels were quantified by ELISA. (*p < 0.01 versus Sham; ^#^*p* < 0.01 versus IRI). Results are expressed as mean ± s.d. (**i**) Kidney tissues were obtained one day after IRI and homogenized to measure ROS formation. (*p < 0.01 versus Sham; ^#^*p* < 0.01 versus IRI). Results are expressed as mean ± s.d. (**j**) Renal tissues were collected one day after IRI and stained by immunohistochemistry to detect MPO-positive cells (the same mice that were sacrificed for the assessment of pathologic damage were used for each group). Original magnification × 400 over five fields. MPO staining for neutrophil infiltration (brown: MPO-positive cells). Bar = 400 μm in all panels. (**k**) Semi-quantitative analysis of MPO positive cells. (*p < 0.01 versus Sham; ^#^*p* < 0.01 versus IRI). Results are expressed as mean ± s.d.

To evaluate the protective effects of TJ-M2010-2 or dual system inhibition on IRI-induced inflammatory responses, we measured NF-κB nuclear translocation, activator protein-1 (AP-1) and cyclooxygenase 2 (COX2) expression, reactive oxygen species (ROS) and cytokine production and myeloperoxidase (MPO) activity. DAMPs released during renal IRI initiate TLR/MyD88 signaling, which leads to NF-κB nuclear translocation^8^. To test whether TJ-M2010-2 inhibited IRI-induced NF-κB activity, we performed an electrophoretic mobility shift assay (EMSA) assay to detect nuclear NF-κB binding to an NF-κB 3′ biotinylated probe (AGTTGAGGGGACTTTCCCAGGC) and found that IRI markedly increased NF-κB activation. TJ-M2010-2 or dual system inhibition treatment remarkably inhibited NF-κB activity (Fig. 3a,b). We next evaluated the inflammatory cytokines AP-1 and COX2. TJ-M2010-2 alone or in combination with MR1 significantly decreased AP-1 and COX2 levels (Fig. 3c,d). To explore how cytokine levels change after IRI, we measured the *in vivo* serum levels of IL-1β, IL-6, TNF-α and interleukin-10 (IL-10). As shown in Fig. 3e–h, TJ-M2010-2 treatment or dual system inhibition remarkably suppressed IL-1β, IL-6 and TNF-α levels compared to the IRI group. Furthermore, IL-10 levels significantly increased in the TJ-M2010-2 and TM groups. Reperfusion is associated with ROS formation, which is responsible for kidney injury^19^,^20^. Thus, we examined ROS levels in renal tissues. As shown in Fig. 3i, TJ-M2010-2 treatment or dual system inhibition significantly decreased ROS production. To evaluate neutrophil infiltration, we measured MPO activity in kidneys one day after IRI. As shown in Fig. 3j,k, TJ-M2010-2 treatment or dual system inhibition significantly reduced renal MPO activity. These results suggest that TJ-M2010-2 shows strong anti-inflammatory effects after IRI.

### TJ-M2010-2 or dual system inhibition attenuates IRI-induced apoptosis

**Figure 4:**
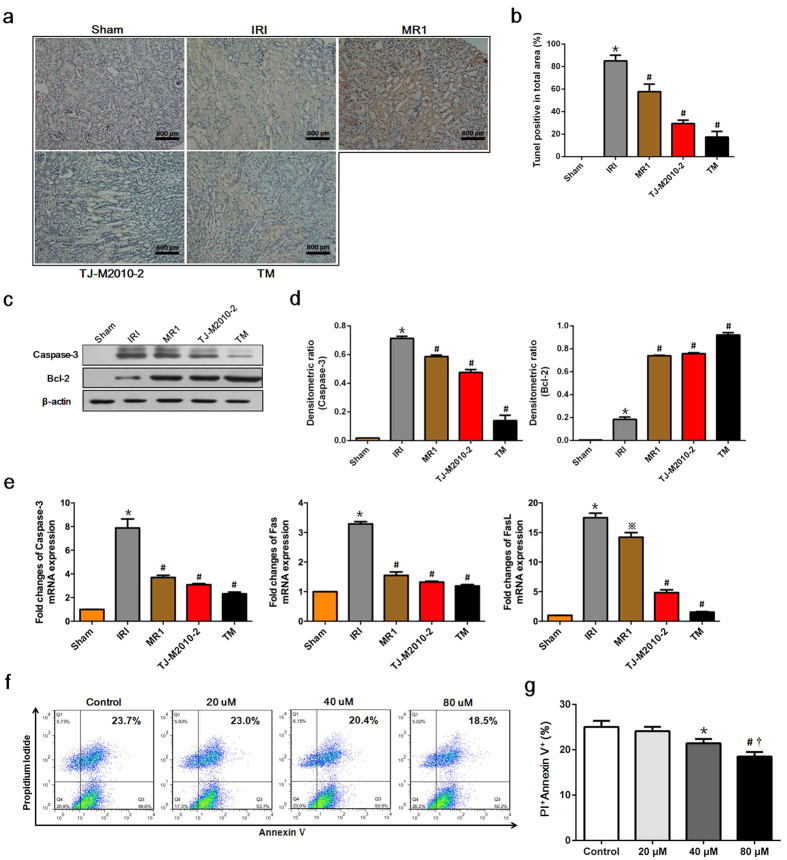
TJ-M2010-2 alone or with MR1 decreases IRI-induced apoptosis. Mice were exposed to IRI for 80 min with uninephrectomy. (**a**) Renal tissues were dissected one day after IRI and the amount of renal apoptosis was analyzed by TUNEL assay (the same mice that were sacrificed for the assessment of pathologic damage were used for each group). Original magnification × 200 over five fields. Bar = 800 μm in all panels. (**b**) Semi-quantitative analysis of apoptotic area. The level of apoptosis was expressed as the percent TUNEL-positive area from total area. (*p < 0.01 versus Sham; ^#^*p* < 0.01 versus IRI). Results are expressed as mean ± s.d. (**c**) Total proteins were extracted from the kidney one day after reperfusion. Caspase-3 and Bcl-2 protein levels were analyzed by Western Blot. (one of three independent experiments). (**d**) Densitometric analysis of Western Blot results. The density of each lane of β-actin was divided by that of Caspase-3 and Bcl-2 (*p < 0.01 versus Sham; ^#^*p* < 0.01 versus IRI). Results are expressed as mean ± s.d. (**e**) RNA was extracted from kidneys one day after IRI. Caspase-3, Fas and FasL mRNA levels were measured by Real-time PCR. (*p < 0.0001 versus Sham; ^#^*p* < 0.0001 versus IRI; ^※^*p* < 0.001 versus IRI). Results are expressed as mean ± s.d. (**f**) HK-2 cells were pre-treated with TJ-M2010-2 (20 μM, 40 μM, 80 μM) and subjected to transient ischemia followed by re-oxygen. HK-2 cells were then stained with annexin V and PI. (one of three independent experiments). (**g**) Quantitative analysis of the results of FCM. (*p < 0.01 versus Control; ^#^*p* < 0.05 versus 40 μM; ^**†**^*p* < 0.01 versus 20 μM). Results are expressed as mean ± s.d.

To explore the anti-apoptotic effect of TJ-M2010-2, we performed a terminal deoxynucleotidyl transferase dUTP nick end labeling (TUNEL) assay, analyzed Caspase-3 and B cell lymphoma-2 (Bcl-2) expression in kidney tissue and determined whether TJ-M2010-2 protected proximal tubular cells against ischemia-induced cell death. IRI induced apoptosis in large areas all over the kidney, while TJ-M2010-2 or dual system inhibition limited apoptosis to small and scattered areas mainly localized to the junction of the cortex and medulla after IRI (Fig. 4a,b). As shown in Fig. 4c–e, TJ-M2010-2 treatment or dual system inhibition markedly decreased Caspase-3 protein and mRNA levels and Fas and FasL mRNA levels. In contrast, Bcl-2 protein levels increased. Furthermore, TJ-M2010-2 treatment reduced human kidney 2 (HK-2) cell apoptosis, with significant protection at 80 μM (Fig. 4f,g). These results demonstrate that TJ-M2010-2 has a strong effect on relieving IRI-induced apoptosis by inhibiting Caspase-3 expression, promoting increased Bcl-2 and strongly protecting tubular cells against IRI.

### TJ-M2010-2 inhibits DC maturation *in vitro* and decreases DC-mediated T-cell proliferation

**Figure 5:**
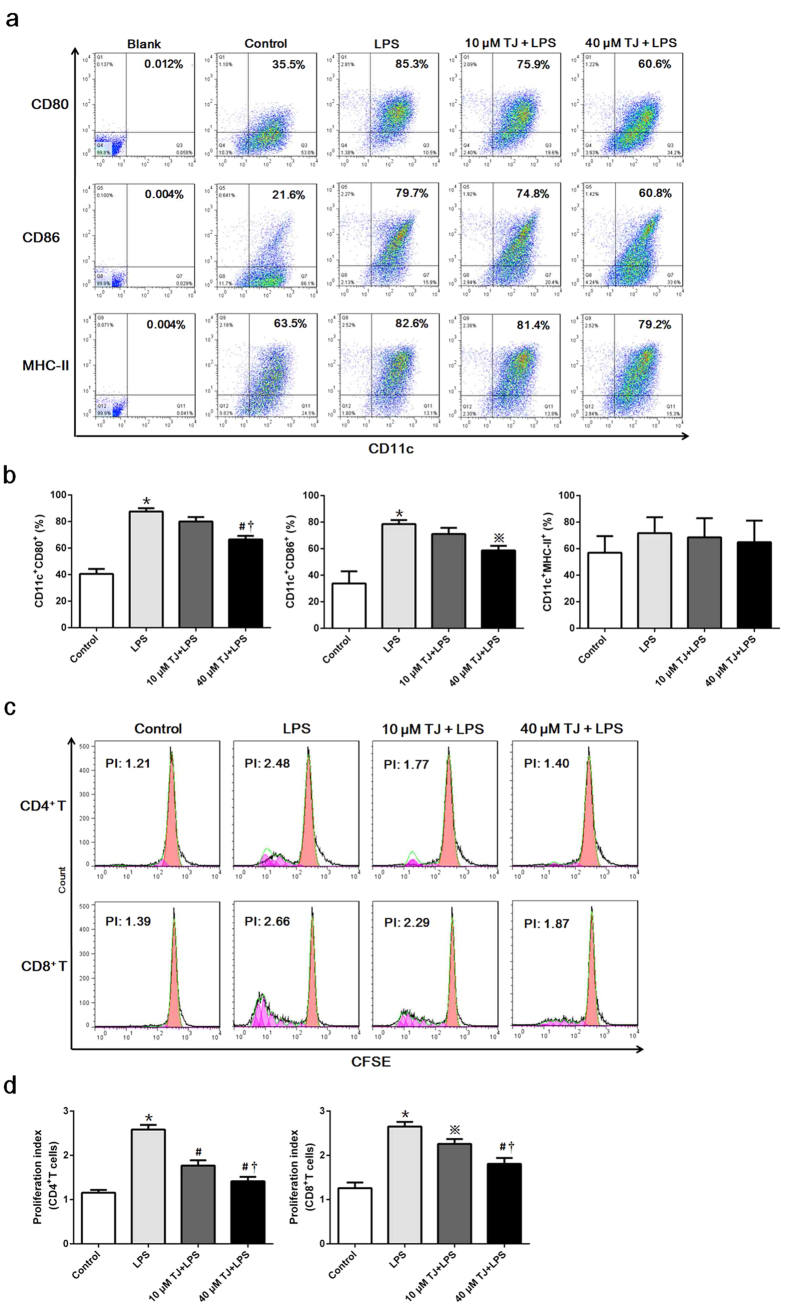
TJ-M2010-2 interferes with DC maturation and decreases T cell proliferation. (**a**) Bone marrow cells from BALB/c mice were cultured with GM-CSF and IL-4 to induce the production of BMDCs. Seven days later, DCs were incubated with TJ-M2010-2 for one hour and then stimulated with LPS for 48 h. CD80, CD86 and MHC-II levels were measured by FCM. TJ-M2010-2 inhibited CD80, CD86 and MHC-II levels dose-dependently (one of three independent experiments). (**b**) Quantitative analysis of the results above. (*P < 0.01 versus Control; ^※^*p* < 0.05 versus LPS; ^#^*p* < 0.01 versus LPS; **†***p* < 0.01 versus 10 μM). Results are expressed as mean ± s.d. Blank: Unstained DCs; Control: DCs stained with CD80, MHC-II, CD86 and CD11c antibodies in the absence of intervention. (**c**) Lymphocytes as responder cells were obtained from C57BL/6 mice and stained with CFSE. BMDCs as stimulator cells were derived from BALB/c mice and treated with LPS with or without TJ-M2010-2. The lymphocytes were co-cultured with BMDCs at a 10:1 ratio for four days, and the proliferation of CD4^+^ and CD8^+^ T cells was measured by FCM. The proliferation index (PI) was regarded as the parameter (one of three independent experiments). PI is the total number of divisions divided by the number of cells that went into division. (**d**) Quantitative analysis of the results from MLR. (^*^p < 0.01 versus Control; ^※^*p* < 0.05 versus LPS; ^#^*p* < 0.01 versus LPS; ^**†**^*p* < 0.01 versus 10 μM). Results are expressed as mean ± s.d. Control: CD4^+^/CD8^+^ T-cells stained with CFSE and co-cultured with DCs in the absence of intervention.

DCs play a critical role in immune response initiation after IRI^21^,^22^. To determine whether TJ-M2010-2 affected DCs maturation and subsequent T-cell proliferation, we tested the inhibitory effects of TJ-M2010-2 on *in vitro* lipopolysaccharides (LPS)-induced DC maturation and T-cell proliferation in mixed lymphocyte reaction (MLR) system. LPS treatment increased DC expression of CD80, CD86 and MHC-II. However, TJ-M2010-2 inhibited DC expression in a dose-dependent manner (Fig. 5a), and 40 μM TJ-M2010-2 significantly inhibited DC maturation (Fig. 5b). CD4^+^ and CD8^+^ T-cell proliferation significantly increased in co-culture with LPS-stimulated DCs and dose-dependently decreased in co-culture with LPS-stimulated DCs pretreated with TJ-M2010-2 (Fig. 5c,d). Furthermore, the concentration of TJ-M2010-2 used in this study did not directly influence the cell viability of DCs and lymphocytes (see Supplementary Fig. S2). These results demonstrate that TJ-M2010-2 inhibits DC maturation and effectively inhibits DC-induced proliferation of CD4^+^ and CD8^+^ T-cells.

### TJ-M2010-2 attenuates renal fibrosis after IRI and prevents EMT *in vitro*

**Figure 6:**
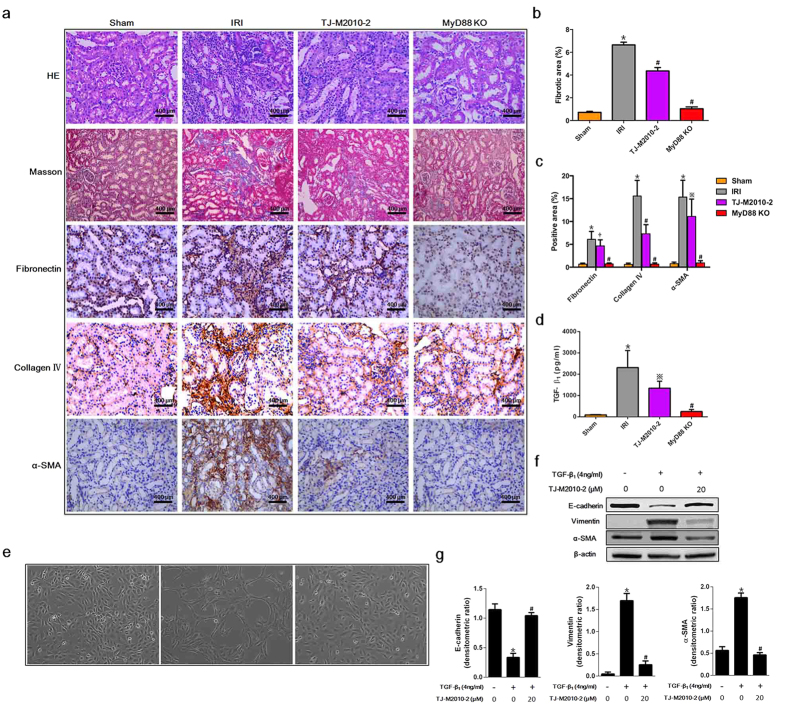
TJ-M2010-2 attenuates IRI-induced renal fibrosis and EMT. Mice were exposed to IRI for 60 min without nephrectomy. (**a**) Left renal tissues were collected 28 days after IRI and stained with Hematoxylin & Eosin, Masson’s trichrome staining, fibronectin, collagen IV and α-SMA (six mice were sacrificed for each group). Original magnification × 400 over five fields. Bar = 400 μm in all panels. (**b**) Semi-quantitative analysis of the fibrotic area using Masson’s trichrome staining. (*p < 0.0001 versus Sham; ^#^*p* < 0.0001 versus IRI). Results are expressed as mean ± s.d. (**c**) Semi-quantitative analysis of positive areas for fibronectin, collagen IV and α-SMA. (^*^p < 0.0001 versus Sham; ^**†**^*p* < 0.05 versus IRI; ^※^*p* < 0.01 versus IRI; ^#^*p* < 0.0001 versus IRI). Results are expressed as mean ± s.d. (**d**) Serum samples were collected 28 days after IRI (three of six mice sacrificed for renal function measurement for each group). TGF-β_1_ levels were quantified by ELISA. (*p < 0.0001 versus Sham; ^※^*p* < 0.01 versus; ^#^*p* < 0.0001 versus IRI). Results are expressed as mean ± s.d. (**e**) Normal HK-2 cells are shown (left, Control group). HK-2 cells were treated with TGF-β_1_ (4 ng/ml) for 72 h (middle, TGF-β_1_ group). HK-2 cells were pre-treated with TJ-M2010-2 for 30 min and then TGF-β_1_ was given as previously described (right). One of three independent experiments is shown. Original magnification × 100. (**f**) Total protein was extracted after TJ-M2010-2 and TGF-β_1_ treatment. E-cadherin, vimentin and α-SMA protein levels were analyzed by Western blot. (one of three independent experiments). (**g**) Densitometric analysis of Western Blot results. The density of β-actin in each lane was divided by that of E-cadherin, vimentin or α-SMA (^*^p < 0.0001 versus Control group; ^#^*p* < 0.0001 versus TGF-β_1_ group). Results are expressed as mean ± s.d.

To evaluate the protective effects of TJ-M2010-2 on renal fibrosis and its mechanism 28 days after surgery, we examined renal interstitial fibrosis, collagen deposition, serum transforming growth factor-β_1_ (TGF-β_1_) and EMT in HK-2 cells. As shown in Fig. 6a–c, the TJ-M2010-2 group showed less tubular atrophy and inflammatory cell infiltration compared to the IRI group. TJ-M2010-2 attenuated fibronectin expression, which is a fibroblast marker. Furthermore, Masson’s trichrome staining and collagen IV staining indicated that renal interstitial fibrosis and collagen deposition were significantly decreased upon TJ-M2010-2 treatment compared to the IRI group. We also examined the level of α-smooth muscle actin (α-SMA), a myofibroblast activation marker, which was down-regulated upon TJ-M2010-2 administration. We measured TGF-β_1_ serum levels, as it is known to play a vital role in renal interstitial fibrosis. TJ-M2010-2 treatment significantly attenuated TGF-β_1_ levels compared to the IRI group (Fig. 6d). Moreover, the MyD88 KO group exhibited the best protective effects among the Sham, IRI and TJ-M2010-2 groups (Fig. 6a–d). We next evaluated the EMT process in HK-2 cells. As shown in Fig. 6e, normal HK-2 cells showed a cobblestone morphology, and TGF-β_1_ treatment altered the morphology to an elongated shape with filopodial extensions. TJ-M2010-2 administration restored the normal cobblestone morphology. Furthermore, we measured E-cadherin expression and the mesenchymal cell markers vimentin and α-SMA. As shown in Fig. 6f,g, TGF-β_1_ administration significantly decreased E-cadherin expression and increased vimentin and α-SMA expression. However, TJ-M2010-2 incubation reversed the effects of TGF-β_1._ Therefore, TJ-M2010-2 ameliorates renal fibrosis by decreasing TGF-β_1_ levels and inhibiting EMT.

### TJ-M2010-2 or dual system inhibition down-regulates the TLR/MyD88 signaling pathway

**Figure 7:**
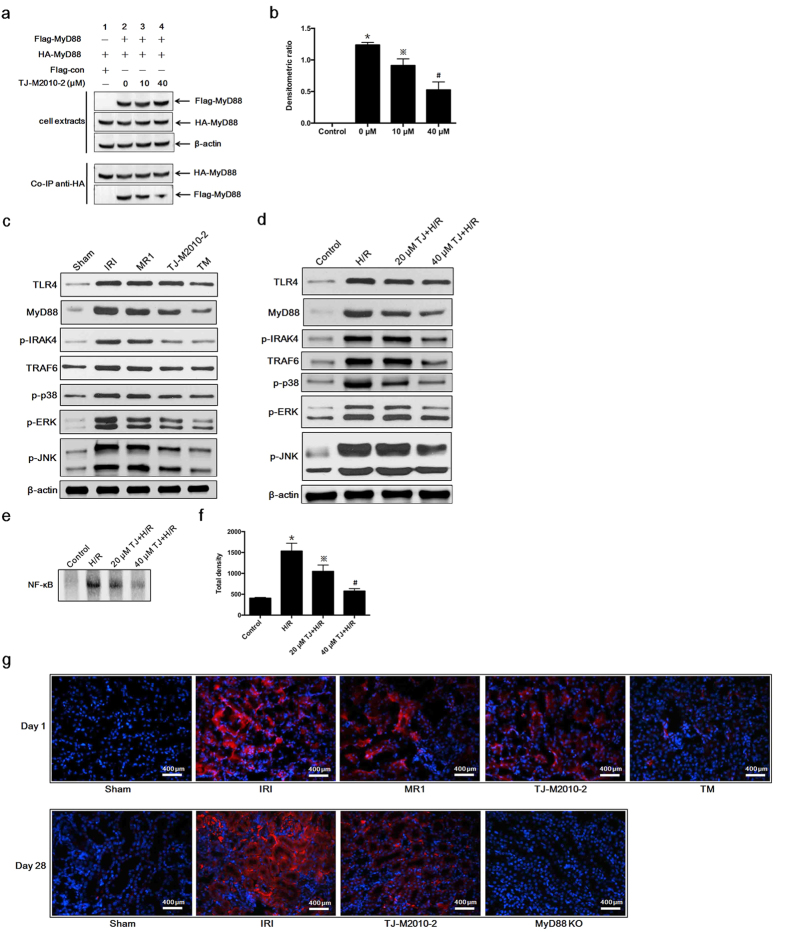
TJ-M2010-2 down-regulates the TLR/MyD88 signaling pathway. (**a**) HK-2 cells were co-transfected with HA-MyD88/Flag-MyD88 or HA-MyD88/Flag-con. Co-IP assays were performed the same as with HEK293T cells (one of three independent experiments). (**b**) Densitometric analysis of Co-IP assays. The density of each HA-MyD88 lane was divided by that of Flag-MyD88 (^*^p < 0.0001 versus Control; ^※^*p* < 0.01 versus 0 μM; ^#^*p* < 0.0001 versus 0 μM). Results are expressed as mean ± s.d. (**c**) Mice were exposed to IRI for 80 min with uninephrectomy (three mice were sacrificed for each group). Total proteins were extracted from kidney one day after IRI. TLR4, MyD88, p-IRAK4, TRAF6, p-p38, p-JNK and p-ERK protein levels were analyzed by Western Blot (one of three independent experiments). (**d**) Total proteins were extracted from HK-2 cells exposed to H/R injury. TLR4, MyD88, p-IRAK4, TRAF6, p-p38, p-JNK and p-ERK protein levels were analyzed by Western Blot (one of three independent experiments). (**e**) Nuclear proteins were extracted from HK-2 cells exposed to H/R injury. EMSA assay was used to detect NF-κB activity (one of three independent experiments). (**f**) Densitometric analysis of the NF-κB band in EMSA. (^*^p < 0.0001 versus Control; ^※^*p* < 0.01 versus H/R; ^#^*p* < 0.0001 versus H/R). Results are expressed as mean ± s.d. (**g**) Three mice were sacrificed for each group. Renal tissues from short-term observation (80 min with uninephrectomy, day 1) and long-term observation (60 min without nephrectomy, day 28) were stained with MyD88 (red) and nucleus (blue).

**Figure 8:**
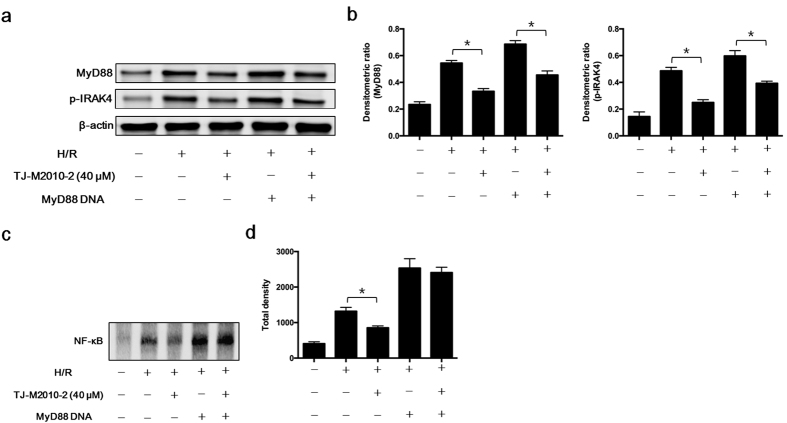
MyD88 DNA reverses the protective effect of TJ-M2010-2. HK-2 cells were transfected with or without MyD88 DNA followed by H/R injury. (**a**) Total proteins were extracted from HK-2 cells. MyD88 and p-IRAK4 protein levels were analyzed by Western Blot (one of three independent experiments). (**b**) Densitometric analysis of Western Blot results. (^*^p < 0.0001). Results are expressed as mean ± s.d. (**c**) Nuclear proteins were extracted from HK-2 cells. An EMSA assay was used to detect NF-κB activity (one of three independent experiments). (**d**) Densitometric analysis of the NF-κB band in EMSA. (^*^p < 0.05). Results are expressed as mean ± s.d.

Because the TLR/MyD88 signaling pathway had a critical role in initiating renal IRI, we evaluated the effect of TJ-M2010-2 on this pathway. As shown in Fig. 7a,b, TJ-M2010-2 inhibited MyD88 homodimerization in HK-2 cells. Furthermore, TJ-M2010-2 down-regulated TLR4, MyD88, phospho-interleukin-1 receptor-associated kinase 4 (p-IRAK4), tumor-necrosis factor receptor associated factor 6 (TRAF6), p-p38, p-JNK and p-ERK levels and NF-κB nuclear translocation in renal tissues and HK-2 cells (Fig. 7c–f). We performed immunofluorescence of MyD88 in renal tissues. TJ-M2010-2 decreased renal MyD88 expression in both acute and chronic IRI (Fig. 7g). We next over-expressed MyD88 by transfecting HK-2 cells with MyD88 DNA. MyD88-overexpressing HK-2 cells exhibited much stronger TLR/MyD88 signaling effects compared to untransfected cells in the presence or absence of TJ-M2010-2. For example, we observed higher MyD88 and p-IRAK4 protein levels and increased NF-κB nuclear translocation (Fig. 8). Therefore, TJ-M2010-2 ameliorated renal IRI by inhibiting the TLR/MyD88 signaling pathway, but MyD88 over-expression reversed the protective effects of TJ-M2010-2.

### Body weight and kidney weight measurements 28 days after surgery

To evaluate the degree of renal atrophy 28 days after IRI, we measured the body weight and kidney weight among different groups. As shown in Table 1, the ratio of left kidney weight to body weight and the ratio of left kidney weight to right kidney weight were significantly greater in the TJ-M2010-2 group compared to the IRI group, while the ratio in the MyD88 KO group was similar to the Sham group. Therefore, TJ-M2010-2 administration restored the weight loss and ameliorated renal atrophy.

## Discussion

Although the innate immune system initiates the immune responses and plays a critical role in IRI, there is not currently a drug available that targets the innate immune system or that prevents renal IRI. We designed and synthesized TJ-M2010-2 to inhibit innate immunity, and we investigated its anti-IRI effects and underlying mechanisms in a renal I/R mouse model. TJ-M2010-2 prolonged the survival rate, protected renal function and ameliorated the inflammatory responses and apoptosis in the short-term by inhibiting the TLR/MyD88 signaling pathway. TJ-M2010-2 also showed a long-term effect by attenuating renal fibrosis via EMT inhibition.

TLR/MyD88 signaling pathway plays a dominant role in mediating kidney damage induced by I/R^23^. During renal IRI, TLR expression, the indication of innate immune response activation, dramatically increases. Several TLRs have been widely studied in renal IRI, including TLR2 and TLR4^24^,^25^,^26^,^27^. Leemans *et al.* found that TLR2 played a crucial role in the induction of inflammatory injury in renal I/R^25^. Li *et al.* showed that the inhibition of TLR4/MyD88 signaling protected mice against ischemia induced acute kidney injury^26^. All TLRs, except TLR3, need MyD88 as their adaptors^8^. Most TLR ligands bind to individual receptors to promote MyD88 homodimerization and then MyD88 recruits IRAK4, IRAK1, IRAK2, TRAF6 to induce inflammatory responses by activating NF-κB and MAPKs^8^,^16^. In addition, several receptors and adapters in the TLR/MyD88 signaling pathway of innate immunity contain a TIR domain, which contains several highly conserved residues. Some of these proteins include TLRs, MyD88, TIR adaptor protein (TIRAP), TIR-domain-containing adapter protein inducing interferon-β (TRIF) and TRIF-related adapter molecule (TRAM)^28^. Therefore, we synthesized TJ-M2010-2 based on the MyD88 TIR domain structure. As we have shown, TJ-M2010-2 acts on αA, αE, βC, βD, DD loop, EE loop and the Poc site residue I179, which alters MyD88 configuration, electron cloud distribution and stability to influence TIR:TIR domain interactions. Our results show that TJ-M2010-2 blocks TLR/MyD88 signaling by affecting MyD88 homodimerization. Further studies to determine whether TJ-M2010-2 also influences MyD88 heterodimerization with TLRs and MAL, especially with TLR2 and TLR4, are required.

In I/R, DCs participate in the early phase of IRI and play a central role in mediating renal I/R damage^21^,^29^ by binding kidney endothelium infiltrating the kidneys^30^, and priming the adaptive immune response. The immune response is primarily controlled by TLR/MyD88 signaling in DCs^31^. After TLR engagement in DCs by DAMPs, MyD88 homodimerization leads to downstream signal transduction and subsequent NF-κB nuclear translocation^32^,^33^. Thus, the TLR/MyD88/NF-κB signaling in DCs plays a critical role in the induction of renal IRI and the blockade of that signaling by TJ-M2010-2 effectively protects against I/R induced AKI. Meanwhile, TJ-M2010-2 demonstrated strong inhibitory effects on TLR/MyD88 signaling in HK-2 cells that had undergone H/R injury and mouse renal tissues exposed to I/R injury. We also observed robust and multiple protective effects against renal IRI, for instance, decreased T cell proliferation and ROS production, the rescue of tubular apoptosis *in vivo* and the protection of HK-2 cells against apoptosis *in vitro*, and the decrease of pro-inflammatory cytokines, including IL-1β, IL-6 and TNF-α and the increase of anti-inflammatory cytokine IL10 accompanied with decreased neutrophil infiltration.

Although TLR/MyD88 signaling plays a dominant role in initiating the immune response during renal IRI, there are still other signaling pathways that participate in activating the adaptive immune response. Therefore, it is not adequate to target only innate immunity and we must also block adaptive immunity to achieve maximum protection against AKI. Thus, we designed a dual system inhibition protocol against the innate and adaptive immune systems simultaneously by applying TJ-M2010-2 with the CD154 antagonist MR1. Some studies have shown that CD154 plays an important role in IRI in the kidney^34^, heart^35^ and liver^36^,^37^. In our study, the MR1 treatment group exhibited higher survival rates and better renal function compared to the mice in the Sham group, though the protective effects of MR1 were not as strong as those of TJ-M2010-2. To our surprise, 100% of the mice in the TM group survived and had the best renal function, as assessed by Cr and BUN levels. These results verify that targeting both immune systems simultaneously is important when designing new and better therapeutic strategies to prevent AKI.

Renal fibrosis is the final result of CKI in IRI^38^. EMT is an important pathological pathway to tubulointersitial fibrosis development, and TGF-β_1_ is the critical profibrotic factor in this process^39^. In this study, TGF-β_1_ initiated EMT *in vitro,* and TJ-M2010-2 treatment blocked EMT. Although some studies have reported that MyD88 inhibition relieved renal fibrosis, the exact mechanism is not clear. We found that the anti-fibrotic effect of TJ-M2010-2 was associated with the amelioration of TGF-β_1_-induced EMT. Therefore, MyD88 plays a critical role in EMT leading to renal fibrosis. MyD88 is a potential novel clinical target to reduce chronic kidney injury.

In summary, our results demonstrate the powerful protection of TJ-M2010-2 against acute and chronic renal IRI by inhibiting the TLR/MyD88 signaling pathway. The dual system inhibition strategy provides new insight into the approaches for treating and preventing AKI. The prevention of renal fibrosis through MyD88 inhibition results from the reduction of TGF-β_1_-induced EMT.

## Methods

### Animals

Male BALB/c (H-2^d^) and C57BL/6 (H-2^b^) mice (six-to-eight weeks old) were purchased from HFK Bio-Technology Co. Ltd. (Beijing, China). BALB/c MyD88^−/−^ (H-2^d^) mice were generously provided by Dr. Maria-Luisa Alegre (University of Chicago, Chicago, IL, USA.). All animals were housed in specific pathogen-free facilities and maintained under controlled conditions (22 °C, 55% humidity and 12 h day/night cycle) at Huazhong University of Science and Technology, Wuhan, China. All animal experiments were approved by the Institutional Animal Care and Use Committee at Tongji Hospital. The methods were carried out in accordance with the approved guidelines.

### Molecular docking of TJ-M2010-2 with the MyD88 TIR domain

The molecular docking method, which was accomplished by the Cerius2 LigandFit (Accelrys Inc. San Diego, CA, USA) software, was applied to study the interaction site of TJ-M2010-2 with the MyD88 TIR domain.

### Induction of renal IRI model

Renal IRI model was performed as the method reported with some modifications^40^. Briefly, 80 min ischemic time with uninephrectomy was used for short-term observation (less than 7 days) and 60 min without nephrectomy was used for long-term observation (28 days). Day 0 was the day of the operation. The mice were divided into six groups, as follows: (1) Sham group: BALB/c mice underwent sham operation; (2) IRI group: BALB/c mice underwent IRI; (3) MR1 group: BALB/c mice were pretreated with MR1 and then underwent IRI; (4) TJ-M2010-2 group: BALB/c mice were pretreated with TJ-M2010-2 alone and then underwent IRI; (5) MyD88 KO group: BALB/c MyD88^−/−^ mice underwent IRI; (6) TM group: BALB/c mice were pretreated with TJ-M2010-2 and MR1 and then underwent IRI.

### Reagents and administration

TJ-M2010-2 was synthesized at the Academy of Pharmacy, Tongji Medical College, Huazhong University of Science and Technology, Wuhan, China (WIPO Patent, application number: PCT/CN2012/070811). Nuclear magnetic resonance and high performance liquid chromatography were used to analyze the structure and purity of TJ-M2010-2, respectively. The molecular structure and chemical synthesis of TJ-M2010-2 were shown (see Supplementary Fig. S3). Mice were intraperitoneally injected with 50 mg/kg TJ-M2010-2 dissolved in 0.5% carboxymethyl cellulose with or without MR1 (Bio X Cell, West Lebanon, NH, USA) (200 μg/day) on day -2, -1, 0 for short-term administration. Furthermore, TJ-M2010-2 was given to mice from day -2 to day 7 for long-term administration.

### Measurement of blood and renal parameters

Blood samples were collected from the inferior vena cava and centrifuged (7500 g, 10 min) for serum collection on day 1, day 3, day 7 and day 28. Serum Cr and BUN concentrations were measured by the clinical laboratory of Tongji Hospital (Wuhan, China). Serum IL1β, IL6, TNF-α and IL10 of day 1 and TGF-β_1_ of day 28 concentrations were measured by ELISA kits (eBioscience, San Diego, CA, USA) according to the manufacturer’s instructions. ROS of day 1 were measured by ROS assay kit (Beyotime Institute of Biotechnology) according to the manufacturer’s instructions. Body weight and kidney weight were measured on day 28.

### Plasmids and cell culture

Flag-MyD88, HA-MyD88 and Flag-tagged control pcDNA 3.1- (Flag-MyD88, HA-MyD88, Flag-con) were generated by Shanghai GeneChem. Co. Ltd. (Shanghai, China). The Flag-con lacked the MyD88 sequence (NM_002468). Human embryonic kidney 293 T (HEK293T) cells (Cell Bank of the Committee of Type Culture Collection of the Chinese Academy of Sciences, Shanghai, China) were cultured in Dulbecco’s modified Eagle medium supplemented with 10% fetal bovine serum (both from Gibco, Carlsbad, CA, USA) in a 37 °C humidified atmosphere of 5% CO_2_. HK-2 cells (China Center for Type Culture Collection, Wuhan, China) were cultured in RPMI medium 1640 supplemented with 10% fetal bovine serum (Gibco) in a 37 °C humidified 5% CO_2_ atmosphere.

### Co-IP

HEK293T and HK-2 cells were co-transfected with HA-MyD88/Flag-MyD88 or HA-MyD88/Flag-con using Lipofectamine 2000 (Invitrogen, Carlsbad, CA, USA) following the manufacturer’s instructions. The subsequent procedures were performed as the method reported^41^,^42^.

### Generation and intervention of bone marrow-derived dendritic cells (BMDCs)

BMDCs were obtained as the method described with some modifications^43^,^44^. On incubation day 3 and 6, fresh medium containing granulocyte-macrophage colony-stimulating factor (GM-CSF) and IL-4 was added to the plate. On incubation day 7, TJ-M2010-2 was added (0 μM, 10 μM and 40 μM) for 1 h, after which LPS, (1 μg/ml^45^,^46^, Sigma, St. Louis, MO, USA) was added for an additional 48 h. Finally, non-adherent cells were collected and stained with FITC-conjugated anti-CD80, PE-conjugated anti-MHC-II, PE-Cy5-conjugated anti-CD86 and APC-conjugated anti-CD11c (all from eBioscience, San Diego, CA, USA) for analysis by flow cytometry (FCM).

### MLR

The MLR was performed as the method previously described^44^. After four days reaction, the cells were collected and stained with PE-conjugated anti-CD4 and APC-conjugated anti-CD8 (eBioscience). Cell proliferation was analyzed by FCM and the proliferation index (PI).

### Histology, immunohistochemistry and immunofluorescence

Left kidneys were dissected from mice 1 day and 28 day after I/R and fixed in 10% formalin. Formalin-fixed kidneys were embedded in paraffin, and 4 μm sections were stained with Hematoxylin and Eosin (H&E). Tubular necrosis was semi-quantitatively analyzed according to previously reported methodology^47^. Renal MPO, fibronectin, collagen IV and α-SMA was evaluated by immunohistochemistry as the method previously described^40^. Masson’s trichrome staining was used to evaluate renal fibrosis. Twenty-four hours after I/R, cell apoptosis in paraffin sections was analyzed by TUNEL assay with the *in Situ* Cell Death Detection kit, POD (Roche, Basel, Switzerland) according to the manufacturer’s instructions. Histopathology and immunohistochemistry scoring was performed by pathologists blinded to the treatment groups. Frozen (day 1) and formalin-fixed (day 28) kidneys were used to perform immunofluorescence analysis with a MyD88 antibody (Abcam) at 4 °C overnight. A Cy3-conjugated secondary antibody was then added at 37 °C for 50 min. Cell nuclei were stained with DAPI.

### Cell hypoxia/reoxygenation (H/R) injury

HK-2 cells were cultured in a 6-well plate at 7.0 × 10^5^ cells/ml for 24 h. The cells in the H/R group were induced to become transiently ischemic by culturing them in a 37 °C humidified atmosphere with no oxygen and 5% CO_2_ for 5 h, and then cultured under normal conditions for 1 h. The cells in the TJ-M2010-2 group were pre-treated with TJ-M2010-2 (20 μM or 40 μM) for 24 h before H/R injury.

### *In vitro* apoptosis quantification in HK-2 cells

For *in vitro* apoptosis quantification, TJ-M2010-2 was added (20 μM, 40 μM and 80 μM) and incubated for an additional 24 h before H/R injury. Cells were collected and then stained with annexin V and propidium iodide (MultiSciences (Lianke) Biotech Co., Ltd. Hangzhou, China).

### *In vitro* EMT reversal assay in HK-2 cells

HK-2 cells were seeded in a 6-well plate at 1.0 × 10^5^ cells/ml for 24 h to approximately 60% confluence. The medium was then replaced with serum-free medium. TJ-M2010-2 was added (0 μM, 20 μM) to the culture for 30 min, followed by addition of TGF-β_1_ (4 ng/ml) for 72 h. Morphological changes were assessed by phase contrast microscopy and E-cadherin, vimentin and α-SMA expression were evaluated by Western Blot.

### *In vitro* MyD88 overexpression assay in HK-2 cells

HK-2 cells were transfected with HA-MyD88 in the presence or absence of TJ-M2010-2 (40 μM) for 24 h before H/R injury. MyD88 and p-IRAK4 protein levels and NF-κB nuclear translocation were measured.

### Real-time PCR

Total RNA was extracted from renal tissues using Trizol reagent (Invitrogen). Reverse transcription was performed using RevertAid First Strand cDNA Synthesis Kit (Thermo Scientific). Real-time PCR was performed using SYBR^®^ Green Realtime PCR Master Mix (TOYOBO, Japan) with ABI PRISM^®^ 7700. The primer sequences are listed as Supplementary Table S1 online. The results were analyzed using the comparative CT method, and actin was used as the housekeeping gene.

### EMSA

Nuclear proteins were extracted from the left kidney (harvest on day 1) and HK-2 cells with a Nuclear and Cytoplasmic Protein Extraction Kit (Beyotime Institute for Biotechnology). EMSA was performed with the LightShift Chemiluminescent EMSA Kit (Thermo Scientific, Waltham, MA, USA) according to the manufacturer’s protocol.

### Western blot analysis

On day 1, total protein was extracted from renal tissue homogenate. The protein of HEK-293 T and HK-2 cells were lysed with IP lysis buffer containing the protease inhibitor PMSF (both from Beyotime Institute of Biotechnology). Western blot was performed as the method previously described with some modifications^42^. The membrane was immunoblotted with antibodies against Caspase-3, Bcl-2, TLR4, MyD88, p-IRAK4, AP-1, Cox2, p-p38, p-JNK, p-ERK, HA, Flag (Cell Signaling Technology, Danvers, MA, USA), TRAF6, E-cadherin, vimentin, α-SMA (Abcam), β-actin (Beyotime Institute of Biotechnology) and detected with horseradish peroxidase-conjugated secondary antibodies and ECL A/B reagents (Beyotime Institute of Biotechnology).

### Statistical Analysis

Data are presented as mean ± standard deviation (s.d.). Survival rates were compared with a log-rank test, and different groups were compared with a Student’s *t*-test or one-way ANOVA, as appropriate. Statistical analysis was performed on Graphpad Prism Software (GraphPad Software, Inc. La Jolla, CA, USA). *P* values < 0.05 were considered statistically significant.

## Additional Information

**How to cite this article**: Zhang, L.-M. *et al.* Pharmacological inhibition of MyD88 homodimerization counteracts renal ischemia reperfusion-induced progressive renal injury in vivo and in vitro. *Sci. Rep.* **6**, 26954; doi: 10.1038/srep26954 (2016).

## Supplementary Material

Supplementary Information

## Figures and Tables

**Table 1:** Body weight and kidney weight, 28 days after IRI.

	Sham (n = 6)	IRI (n = 6)	TJ-M2010-2 (n = 6)	MyD88 KO (n = 6)
Body weight (BW) (g)	25.05 ± 1.75#	30.22 ± 0.43	28.92 ± 0.68	24.43 ± 1.43#
Right kidney weight (RKW) (g)	0.2107 ± 0.0366#	0.3246 ± 0.0099	0.2921 ± 0.0089*	0.2307 ± 0.0189#
RKW to BW ratio	0.0083 ± 0.0008	0.0107 ± 0.0002	0.0102 ± 0.0005	0.0094 ± 0.0004*
Left kidney weight (LKW) (g)	0.1932 ± 0.0250†	0.0846 ± 0.0072	0.1123 ± 0.0131	0.1499 ± 0.0130#
LKW to BW ratio	0.0077 ± 0.0005※	0.0028 ± 0.0003	0.0039 ± 0.0004*	0.0061 ± 0.0004※
LKM to RKW ratio	0.9242 ± 0.0419※	0.2620 ± 0.0211	0.3812 ± 0.0276#	0.6635 ± 0.0604※

Data are mean ± s.d. *P < 0.05, ^#^*P* < 0.01, ^†^*P* < 0.001, ^※^*P* < 0.0001 versus IRI group.
