# Supplementary material for: Pharmacological inhibition of MyD88 homodimerization counteracts renal ischemia reperfusion-induced progressive renal injury in vivo and in vitro
Source: Sci Rep. 2016 Jun 1;6:26954. doi: 10.1038/srep26954 (PMC4887891; doi:10.1038/srep26954)
Supplement: Supplementary Information [file srep26954-s1.pdf]

Title: Pharmacological inhibition of MyD88 homodimerization counteracts renal ischemia reperfusion-induced progressive renal injury in vivo and in vitro

Authors: Li-Min Zhang, Jian-Hua Liu, Cheng-Biao Xue, Ming-Qiang Li, Shuai Xing, Xue Zhang, Wen-Tao He, Feng-Chao Jiang, Xia Lu, Ping Zhou

Supplementary Materials and Methods

Blood Cr and BUN measurements 28 days after IRI

Blood samples were collected from the inferior vena cava and centrifuged (7500 g, 10 min) for serum collection on day 28. Blood Cr and BUN concentrations were measured by the clinical laboratory of Tongji Hospital (Wuhan, China).

Cell viability assay

DCs were cultured and treated as the same way described above with the exception of different concentrations of TJ-M2010-2 (0 μM, 10 μM, 20 μM, 40 μM, 80 μM). DCs were then stained with annexin V and PI. Lymph nodes from C57BL/6 mice were ground and filtered through nylon mesh to collect lymphocytes. The lymphocytes were cultured with CD3 (2 μg/ml), CD28 (1 μg/ml) and TJ-M2010-2 (0 μM, 10 μM, 20 μM, 40 μM, 80 μM) in a 96-well plate with flat bottoms. After three days, lymphocytes were collected and stained with annexin V and PI.

Chemical synthesis of TJ-M2010-2

1. Synthesis of 1-p-tolylpiperazine chloride: Bis (2-chloroethyl) ammonium chloride (24 mmol) and p-toluidine (20 mmol) in n-butyl alcohol (50 ml) were mixed and microwaved for six minutes at 195 W. Sodium carbonate (12 mmol) was added to the reaction and

microwaved for an additional 19 minutes. The solution was filtered and cooled to RT, and filtered double volumetric absolute ethanol was added. Finally, the solution was stewed, filtered, and washed with ethanol and ethyl ether. The crude product was dried and crystallized from ethanol. Vacuum dried, white crystal was collected with a yield of 80%. m.p.:208.2-209.7°C.

2. Synthesis of 2-chloro-N-(4-phenylthiazol-2-yl) acetamide: A solution of 4-phenylthiazol-2-amine (20 mmol) and sodium carbonate (12 mmol) in acetone (20 ml) was cooled to 0°C. After the dropwise addition of cold chloroacetyl chloride (26 mmol) in acetone (10 ml), the reaction mixture was stirred for 3 h at RT and then at 33°C for 4 h. After mixing, the solution was filtered, and the solvent and excess chloroacetyl chloride were removed by distillation. The residue was washed with aqueous sodium bicarbonate (5% w/v), followed by cold water. The crude product was dried and crystallized from ethanol. The result was a yellow granulated product, with a yield of 76%. m.p. 155.2-156.7°C.

3. Synthesis of N-(4-phenylthiazol-2-yl)-2-(4-p-tolylpiperazin-1-yl) acetamide: A mixture of 2-chloro-N-(4-phenylthiazol-2-yl) acetamide (20 mmol), 1-p-tolylpiperazine (20 mmol), absolute ethanol (30 ml), anhydrous sodium carbonate (30 mmol) and a catalytic amount of potassium iodide were combined and microwaved for nine minutes at 195 W. The solvent was removed by distillation, and the residue was treated with 5% sodium bicarbonate solution to remove acid impurities, filtered, washed with water and dried. White crystals were crystallized from 95% ethanol. The general synthesis method is shown in Figure 1a.

UV (CH_3OH) A_{max}/nm 268.1, 235.1;

^{1}H-NMR ($CDCl_3$): *d*2.30 (s, 3H, -CH_3), 2.81 (t, J=6.4 Hz, 4H, a-piperazine), 3.26 (t,

J=6.4 Hz, 4H, b-piperazine), 3.34 (s, 2H, -CH_2-), 6.88 (d, J=7.2 Hz, 2H, phenyl), 7.11 (d, J=7.2 Hz, 2H, phenyl), 7.18 (s, 1H, thiazole C5–H), 6.873-7.868 (m, 5H, phenyl).

^{13}C-NMR ($CDCl_3$): *d*20.45 (-CH_3), 49.85 (2C, -CH_2-, piperazine), 53.72 (2C, -CH_2-, piperazine), 61.15 (-CH_2CO-, piperazine), 107.86 (thiazole C5), 116.75 (2C, -CH-, 4-p-tolyl), 126.09 (2C, -CH-, phenyl), 127.92 (-CH-, phenyl), 128.60 (-CH-, 4-p-tolyl), 128.76 (2C, -CH-, 4-penyl), 129.75 (2C, -CH-, 4-p-tolyl), 134.35 (-CH-, phenyl), 148.78 (-CH-, 4-p-tolyl), 150.14 (thiazole C4), 157.02 (thiazole C2), 168.43 (-CO-).

MS: 392 (15%). HPLC: Ret time 7.663 min, content %: 98.95.

Supplementary Figures and Figure Legends

Supplementary Fig. S1

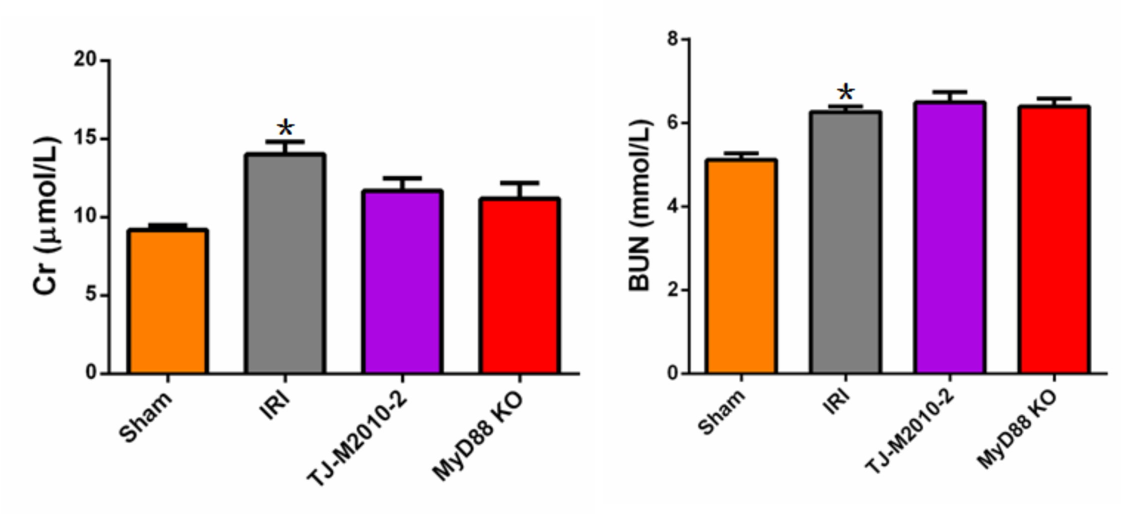

Supplementary Fig. S1. TJ-M2010-2 does not affect serum Cr or BUN levels 28 days after IRI. Blood samples were collected on day 28 to measure serum Cr and BUN levels (six mice were sacrificed for each group). ($^{*}p<0.01$ versus Sham). Results are expressed as mean±s.d..

Supplementary Fig. S2

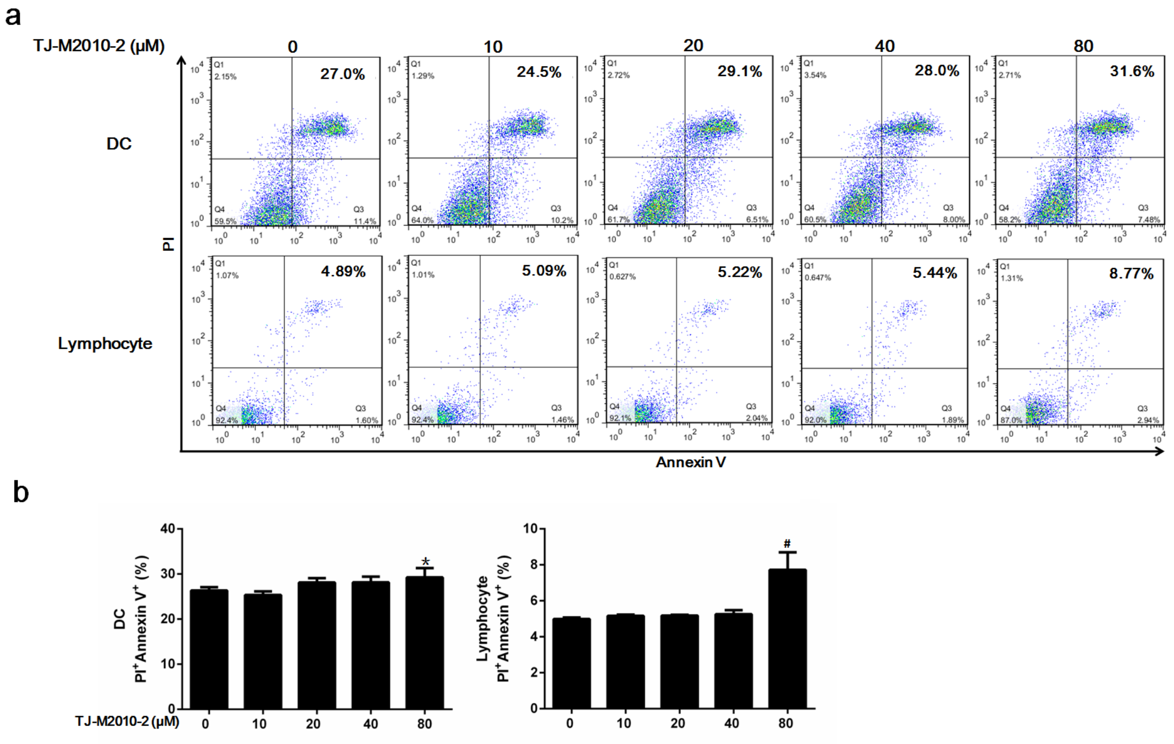

Supplementary Fig. S2. TJ-M2010-2 sustained the cell viability of DCs and lymphocytes. (a) DCs were cultured as the same way mentioned above. Seven days later, DCs were incubated with TJ-M2010-2 (0 μM, 10 μM, 20 μM, 40 μM, 80 μM) for one hour and then stimulated with LPS for 48 h. Then DCs were stained with annexin V and PI. (one of three independent experiments). Lymph nodes from C57BL/6 mice were ground and filtered through nylon mesh to collect lymphocytes. The lymphocytes were cultured with CD3 (2 μg/ml), CD28 (1 μg/ml) and TJ-M2010-2 (0 μM, 10 μM, 20 μM, 40 μM, 80 μM) in a 96-well plate with flat bottoms. Three days later, lymphocytes were collected and stained with annexin V and PI. (one of three independent experiments). (b) Quantitative analysis of the results of FCM. ($^{*}p<0.05$ versus 10 μM; $^{\#}p<0.001$ versus 0 μM, 10 μM, 20 μM, 40 μM). Results are expressed as mean±s.d..

Supplementary Fig. S3

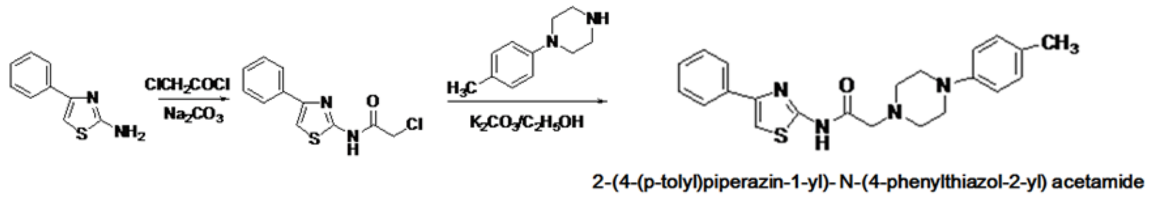

Supplementary Fig. S3. The chemical synthesis of TJ-M2010-2. The chemical synthesis of TJ-M2010-2 and its molecular structure is shown with its full name.

Supplementary Table

Supplementary Table S1. Primer sequences used for real-time PCR

Gene	Primer sequences (5'-3')
Caspase-3	Forward: TGGTGATGAAGGGGTCATTTATG<br>Reverse: TTCGGCTTTCCAGTCAGACTC
Fas	Forward: GCGGGTTCGTGAAACTGATAA<br>Reverse: GCAAAATGGGCCTCCTTGATA
FasL	Forward: TCCGTGAGTTCACCAACCAAA<br>Reverse: GGGGGTTCCCTGTTAAATGGG
β-actin	Forward: CTGAGAGGGAAATCGTGCGT<br>Reverse: CCACAGGATTCCATACCCAAGA
